# Screening of photosensitizers-ATP binding cassette (ABC) transporter interactions *in vitro*

**DOI:** 10.20517/cdr.2024.50

**Published:** 2024-09-21

**Authors:** Shruti Vig, Payal Srivastava, Idrisa Rahman, Renee Jaranson, Anika Dasgupta, Robert Perttilä, Petteri Uusimaa, Huang-Chiao Huang

**Affiliations:** ^1^Fischell Department of Bioengineering, University of Maryland, College Park, MD 20742, USA.; ^2^Laboratory of Cell Biology, Center for Cancer Research, National Cancer Institute, National Institutes of Health, Bethesda, MD 20892, USA.; ^3^Modulight Corporation, Tampere FI-33720, Finland.; ^#^Authors contributed equally.

**Keywords:** Multidrug resistance, ATP-binding cassette (ABC) transporters, photosensitizers, photodynamic therapy

## Abstract

**Aim:** ATP-binding cassette (ABC) transporters are proteins responsible for the efflux of drug molecules from cancer cells, reducing the efficacy of anti-cancer treatments. This study assesses the susceptibility of a panel of clinically used photosensitizers to be transported by ABC transporters *in vitro.*

**Methods:** The involvement of P-glycoprotein (P-gp/ABCB1), breast cancer resistance protein (BCRP/ABCG2), and multidrug resistance-associated protein 1 (MRP1/ABCC1) in the transport of 7 clinically utilized photosensitizers [benzoporphyrin derivative (BPD), temoporfin, redaporfin, talaporfin sodium, rose bengal, methylene blue, and indocyanine green] were investigated using human breast cancer cell lines following well-established protocols. Briefly, parental MCF-7 cells and sublines that overexpress P-gp (MCF-7 TX400), ABCG2 (MCF-7 MX100), or MRP1 (MCF-7/VP) were treated with photosensitizers with and without ABC transporter inhibitors. Intracellular levels of photosensitizers were measured using extraction method and flow cytometry to determine whether the ABC transporters are associated with efflux or uptake of photosensitizers.

**Results:** The ABCG2 inhibitor (fumitremorgin C) and P-gp inhibitor (valspodar) effectively blocked the transport mediated by ABCG2 and P-gp of rose bengal and BPD. Redaporfin showed increased accumulation in the presence of valspodar with flow cytometry. Interestingly, MCF-7/VP cells were found to have reduced intracellular accumulation of rose bengal, which was restored with MRP1 inhibitor (MK571). The cell viability assay showed photodynamic therapy (PDT) resistance with Redaporfin in P-gp-overexpressing cells, BPD in ABCG2- and P-gp-overexpressing cells, and with Rose bengal in ABCG2-, P-gp- and MRP1-overexpressing cells, respectively. However, no change in intracellular retention was observed for other photosensitizers.

**Conclusion:** In summary, our study provided new knowledge that temoporfin, talaporfin sodium, methylene blue, and indocyanine green are not substrates of ABCG2, P-gp, or MRP1. Redaporfin is a substrate for P-gp. BPD is a known substrate of ABCG2 and P-gp. Rose bengal is a substrate of ABCG2, P-gp, and MRP1. The results presented here indicate ABC transporter substrate status as a possible cause for cellular resistance to photodynamic therapy with rose bengal, redaporfin, and BPD.

## INTRODUCTION

Photodynamic therapy (PDT) is a clinically approved treatment modality used to manage neoplastic and nonmalignant diseases^[1]^. PDT utilizes light of specific wavelengths within the 500-800 nm range to stimulate a light-activable compound known as a photosensitizer (PS)^[2]^. The activation of photosensitizer leads to the generation of reactive molecular species such as ^1^O_2_, H_2_O_2_, O^2•-^, ^•^OH, which induces cytotoxicity in adjacent targets^[3,4]^. The cytotoxic effects of PDT are governed by various factors such as intracellular accumulation and distribution of the photosensitizer within cells, the spatial arrangement of light, and the diffusion range of active reactive molecular species (< 0.02 μm)^[2,3,5,6]^.

Several emerging photosensitizers are being investigated for the treatment of tumors^[6,7]^. Many photosensitizers being investigated are chlorins, porphyrins, and dyes. Verteporfin [benzoporphyrin derivative (BPD)], also known as Visudyne, received FDA approval in 2000 for treating wet age-related macular degeneration (AMD)^[8]^. Recent years have seen several clinical trials exploring its efficacy for treating breast cancer^[9,10]^, locally advanced pancreatic cancer^[11]^, melanoma^[12]^, lung cancer^[13]^, and brain tumors^[14]^. Foscan® also known as temoporfin (mTHPC), is approved by the European Medicines Agency (EMA)^[15]^ for treating advanced head and neck cancer and is under clinical trials (Phase I/II) for bile duct carcinoma^[16,17]^ and lung cancer^[18]^ in the US. Laserphyrin (talaporfin sodium) is a mono-L-aspartyl chlorin that was approved in Japan in 2004 for PDT of early-stage lung cancer^[19]^ and is undergoing clinical trials for the treatment of unresectable hepatocellular carcinoma^[20,21]^ and liver metastases of colorectal cancer^[22]^. Redaporfin (LUZ11) received an orphan drug designation from EMA for biliary tract cancer in 2016^[23]^ and is currently undergoing phase I/II trials for advanced head and neck cancer^[24]^. In addition, due to its fluorescence and phototoxic properties, dyes like methylene blue are under investigation as potential photosensitizers for antibacterial and antitumor treatments^[25-27]^. Indocyanine green (ICG) at higher concentrations and light doses has also been used for PDT in several preclinical studies and is currently being assessed for use in treating periodontal disease and in diabetic patients with peri-implantitis^[28,29]^.

The growing interest in the efficacy of photosensitizers for anti-cancer treatments raises concerns regarding the potential drug resistance in cancer cells, thus limiting their therapeutic success. One of the primary mechanisms underlying multidrug resistance involves the overexpression of ATP-binding cassette (ABC) transporters in cancer cells, which has been linked with the chemoresistance phenotype in patients^[30]^. ABC transporters are a family of transporter proteins that are responsible for the efflux of various xenobiotics from the cell to the extracellular space against the concentration gradient through ATP hydrolysis^[31,32]^. Among 48 identified human transporters, breast cancer resistance protein (BCRP) (encoded by ABCG2), P-glycoprotein (P-gp) (encoded by ABCB1), and multidrug resistance-associated protein 1 (MRP1) (encoded by ABCC1) are known to transport a structurally diverse array of cytotoxic compounds including anti-cancer agents and photosensitizers^[33-35]^. ABC transporters potentially decrease the intracellular concentration of substrate photosensitizer to levels insufficient for inducing cell death in tumors subjected to PDT, allowing resistant cells to survive and repopulate the tumor site. Previous research has shown that the ABCG2 transporter can inhibit the intracellular uptake of clinically used photosensitizers, including BPD^[36]^, 5-ALA/PpIX^[37]^, pheophorbide a (PhA)^[38,39]^, and Chlorin e6^[40]^. In addition to ABCG2, BPD is also transported by P-gp but not MRP1 transporter^[36]^. Thus, photosensitizers that are substrates can be effluxed from cancer cells expressing ABC transporters, decreasing the intracellular concentration of photosensitizers below the threshold required to produce a phototoxic response during PDT procedures. While the ABC transporter status of certain photosensitizers is established, numerous upcoming clinically relevant PDT agents are being explored with unknown substrate status for ABC transporters. Understanding the substrate status of these agents is crucial for optimizing PDT outcomes and addressing drug resistance. The structure and clinical applications of the tested agents are summarized in Supplementary Table 1.

In this study, we sought to investigate how P-gp, ABCG2, and MRP1 transporters influence the intracellular accumulation of a panel of clinically relevant photosensitizers using MCF-7 sublines overexpressing respective ABC transporters. Cellular uptake of BPD, temoporfin, talaporfin sodium, redaporfin, ICG, methylene blue, and rose bengal was evaluated using a quantitative intracellular photosensitizer accumulation using extraction method, and flow cytometry method using MCF-7 MX100, MCF-7 TX400, and MCF-7 VP cell lines overexpressing ACBG2, P-gp, and MRP1, respectively. The results obtained from the extraction method were confirmed using qualitative flow cytometry. Our findings confirm that BPD transport is observed only in cells that overexpress ABCG2 and P-gp. Notably, the intracellular accumulation of temoporfin remains unaffected by the tested ABC transporters.

Furthermore, our results suggest that the intracellular levels of other tested photosensitizers were unaffected by ABCG2, P-gp, and MRP1, except for rose bengal. Redaporfin might also potentially interact with the P-gp transporter. Further investigations are warranted to elucidate the structure-activity relationship between ABC transporters and photosensitizers. However, existing literature suggests a correlation between ABC transporter-mediated efflux and the structure of the photosensitizer^[38,41]^.

## METHODS

### Chemicals

BPD was obtained from Adooq Bioscience (CAUSA). Redaporfin and talaporfin sodium were obtained from MedChemExpres (NJ, USA). ICG and temoporfin were obtained from Adooq (CA, USA). Methylene blue and rose bengal were obtained from Sigma-Aldrich (St. Louis, MO, USA). BPD, temoporfin, redaporfin, and ICG were dissolved in DMSO. Talaporfin sodium, rose bengal, and methylene blue were dissolved in phosphate-buffered saline (PBS). The MRP1 inhibitor MK571 was obtained from Sigma (St. Louis, MO). Fumitremorgin C (FTC) and valspodar were obtained from MedChemExpres (NJ, USA).

### Cell culture

The human breast adenocarcinoma cell line MCF-7 and its sublines overexpressing ABC transporters, including the MCF-7 MX100 subline overexpressing ABCG2 (cultured in 100 nM mitoxantrone), the MCF-7 TX400 subline overexpressing P-gp (cultured in 400 ng/mL paclitaxel), and the MCF-7/VP subline overexpressing MRP1 (cultured in 4 µM etoposide), were used in this study^[36]^. The cells were cultured in Eagle’s Minimum Essential Medium (EMEM) (Quality Biological, MD, USA) enriched with 10% fetal bovine serum (FBS; Gibco), 100 µg/mL streptomycin (Lonza), 100 U/mL penicillin, and 0.01 mg/mL insulin (Sigma). Cultures were maintained at 5% CO_2_ and 37 °C. Regular testing confirmed the absence of mycoplasma contamination using the MycoAlert^TM^ PLUS Mycoplasma Detection Kit (Lonza, Basel, Switzerland).

### Western blotting

Western blot analysis was performed to assess ABC Transporter overexpression. MCF-7 sublines (3.0 × 10^5^ cells) were seeded in 35-mm cell culture dishes and allowed to grow for 24 h. Cellular proteins were extracted for western blotting following established protocols^[36]^. 20 µg of proteins from lysates were separated on NuPAGE^TM^ 4%-12% Bis-Tris gels and transferred onto PVDF membranes (Thermo Fisher). Membranes were blocked with 5% milk-containing Tris-buffered saline and polysorbate 20 (TBST) solution for 1 hour, then probed with antibodies for ABCG2 (Kamiya BioMedical MC-177), P-gp (Thermo Fisher MA1-26528), MRP1 (Kamiya BioMedical MC-162), and β-actin antibodies (Cell Signaling 3700). Chemiluminescence generated using SuperSignal West Pico PLUS (Thermo Fisher, MA, USA) was imaged using Azure 500 imager (Azure Biosystems).

### Intracellular photosensitizer accumulation using extraction method

**Figure 1:**
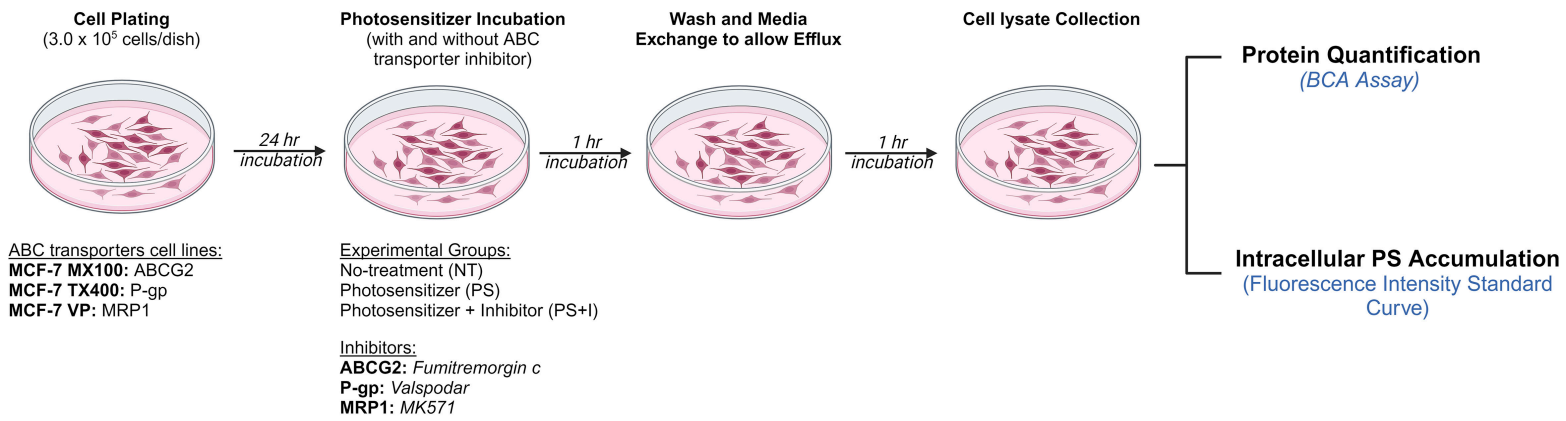
Schematic of the intracellular photosensitizer accumulation quantification (Extraction) method. 3.0 × 10^5^ MCF-7 MX 100, MCF-7 TX400, and MCF-7/VP cells were plated in 35-mm dishes in duplicate and incubated overnight. After 24 h, the cells were incubated with the desired photosensitizer with or without ABC transporter inhibitors (ABCG2: FTC, P-gp: valspodar or MRP1: MK571) for 1 h at 37 °C in 5% CO_2_. Cells were washed with PBS and then incubated for 1 h at 37 °C in a photosensitizer-free medium with or without ABC transporter inhibitors to allow efflux. The cells were subsequently washed and collected for cell lysate preparation. The cell lysates were analyzed using BCA assay for protein quantification. The fluorescence intensity of the experimental groups was recorded using the spectrophotometer at specific Ex/Em wavelengths. Created with BioRender.com. FTC: Fumitremorgin C; PBS: phosphate-buffered saline; ABC: ATP binding cassette; BCA: bicinchoninic acid.

Photosensitizer intracellular accumulation was quantified by adapting the extraction method described previously^[36,42]^. MCF-7 MX100, TX400, and VP cells were plated in 35-mm petri dishes at a cell density of 3.0 × 10^5^ cells per dish to allow overnight culture at 5% CO_2_ and 37 °C. The next day, cells were incubated with media (NT), photosensitizer alone (PS), and photosensitizer with respective ABC transporter inhibitor (10 µM FTC for ABCG2; 3 µg/mL Valspodar for P-gp; 25 µM MK571 for MRP1) (PS+I) at fixed photosensitizer concentration (BPD: 2 μM; ICG: 100 μM; methylene blue: 20 μM; rose bengal: 100 μM; temoporfin: 2 μM; redaporfin: 20 μM; and talaporfin sodium: 200 μM) for 1 h. The stock concentration of the photosensitizer was measured by recording the absorbance spectrum of the agent using the spectrophotometer and extrapolating the concentration with molar extinction coefficients [Supplementary Table 2]. After 1 h, the cells were washed twice with PBS (Corning, USA) and incubated with media for 1 h for efflux. The PS+I group was incubated with an inhibitor during the second incubation to ensure transporter inhibition. To quantify the intracellular photosensitizer uptake, cells were washed with PBS and then lysed in RIPA buffer for 30 min (Thermo Fisher Scientific, USA). A spectrophotometer (Synergy Neo2; Biotek, VT, USA) was utilized to acquire fluorescence signal of photosensitizers [BPD: 435/700 nm (Ex/Em); ICG: 710/810 nm; methylene blue: 610/688 nm; rose bengal: 520/575 nm; temoporfin: 422/660 nm; redaporfin: 510/750 nm; and TS: 398/650 nm]. Bicinchoninic acid (BCA) Protein Assay (ThermoFisher Scientific) was used to determine protein concentration in mg/mL. Intracellular photosensitizer concentrations were quantified using appropriate standard curves [Supplementary Figure 1] and then normalized to total protein concentration as determined by the BCA assay. The experimental protocol for the extraction method is summarized in Figure 1. All experiments were performed at least three times in duplicate (N ≥ 3).

### Flow cytometry

Flow cytometry studies followed previously established protocols^[36,41]^. In brief, MCF-7 cells and sublines (3.0 × 10^5^ cells/dish) were treated with the same concentration of the desired photosensitizer and incubation time points as the extraction studies (i.e., BPD, methylene blue, rose bengal, ICG, temoporfin, and talaporfin sodium). Subsequently, the cells were rinsed with cold PBS before flow cytometry analysis. A fluorescence-activated cell sorting (FACS) flow cytometer (BD FACSCelesta, BD Biosciences) was used for sample measurement. The fluorescence emissions of different photosensitizers were detected using specific lasers and filters: BPD (Ex/Em: 435/690 nm) was detected with a 405 nm laser and a PerCP-Cy5-5 filter (LP670 nm). Temoporfin (Ex/Em: 422/660 nm) and Talaporfin sodium (Ex/Em: 398/650 nm) were observed with a 405 nm laser and BV650 filter (Ex 405 nm / Em 650 ± 30 nm). Rose bengal (Ex/Em: 520/545 nm) was detected with a 488 nm laser and PE filter (Ex 488 nm / Em 575 ± 25 nm). Methylene blue (Ex/Em: 610/688 nm) was evaluated using a 640 nm laser and APC filter (Ex 640 nm / Em 670 ± 30 nm). ICG (Ex/Em: 730/810 nm) and Redaporfin (Ex/Em: 510/750 nm) were studied with a 405 nm laser and BV786 filter (Ex 405 nm / Em 786 ± 60 nm). All experimental conditions were replicated at least three times (*N* ≥ 3). For each flow cytometry analysis, 10,000 events were recorded. FlowJo V10 software was used to analyze gated single-cell populations.

### Cell viability assays

Briefly, 1.0 × 10^4^ cells were plated in 96-well black-walled clear well plates (Southernlabware, GA) and allowed to grow overnight. The next day, test photosensitizer was added to the cells at varying concentrations (200 μL/well) and allowed to incubate in the dark for 24 h at 37 °C. Each test concentration was treated in triplicate. On Day 2, the wells were washed with 1× PBS and replaced with fresh media. Following media exchange, the cells were exposed to a set light dose of 5 J/cm^2^ at excitation wavelengths for respective photosensitizers (BPD: 50 mW/cm^2^, 690 nm; RB: 20 mW/cm^2^, 520 nm; Redaporfin: 20 mW/cm^2^, 520 nm; MB: 50 mW/cm^2^, 665 nm). Cell viability was measured 24 h post-light activation using Cell-Titer Glo^®^ 2.0 luminescent viability assay (Promega Corporation, USA) using the vendor’s protocol. Cell viability was recorded at twelve different testing concentrations per photosensitizer. All experimental conditions were replicated at least three times in triplicates (*N* ≥ 3).

### Statistical analyses

All experiments were performed at least in triplicates. Results are shown with mean ± the standard error of the mean (SEM). Statistical analyses were performed using GraphPad Prism software. One-way analysis of variance (ANOVA) statistical tests and appropriate post hoc analyses were applied to avoid type I errors.

## RESULTS

### ABC transporter overexpression in MCF-7 breast cancer sublines

**Figure 2:**
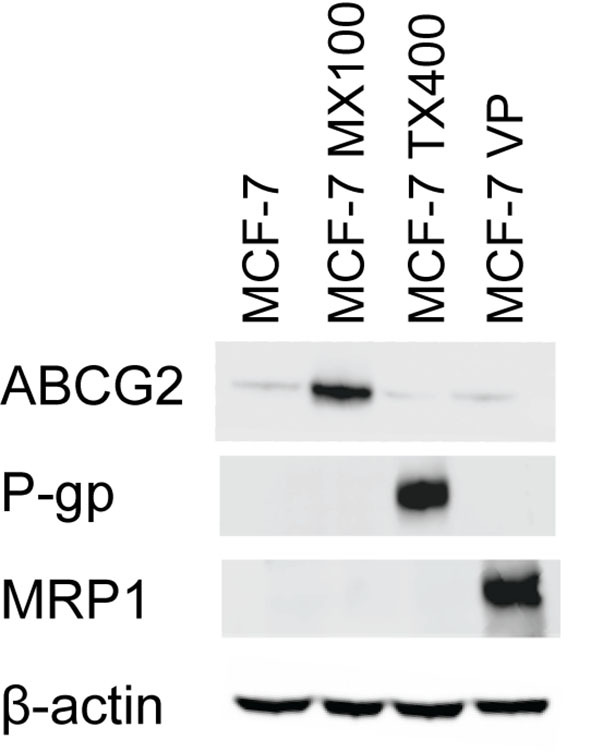
Western blot analysis of ABGC2, P-gp, and MRP1 expression in MCF-7 (parent cell line), MCF-7 MX100, MCF-7 TX400, and MCF-7/VP cells. Whole-cell extracts were collected and analyzed using Western blot. 20 µg of the whole cell extract was loaded in each lane. β-Actin was used as a loading control. ABC transporter selective cell lines show increased expression of respective transporters compared to the parental (control) cell line. ABC: ATP binding cassette.

To assess the impact of ABC transporter on the accumulation of photosensitizers, uptake studies using extraction and flow cytometry methods were performed with MCF-7 breast cancer sublines overexpressing ABCG2, P-gp, and MRP1. Western blot analysis confirmed the overexpression of ABCG2, P-gp, and MRP1 in MCF-7 MX100, MCF-7 TX400, and MCF-7/VP cell lines, respectively. MCF-7 parental cells exhibit no expression of the transporter proteins [Figure 2].

### Quantifying ABC transporter-mediated intracellular accumulation of photosensitizers in MCF-7 sublines

**Figure 3:**
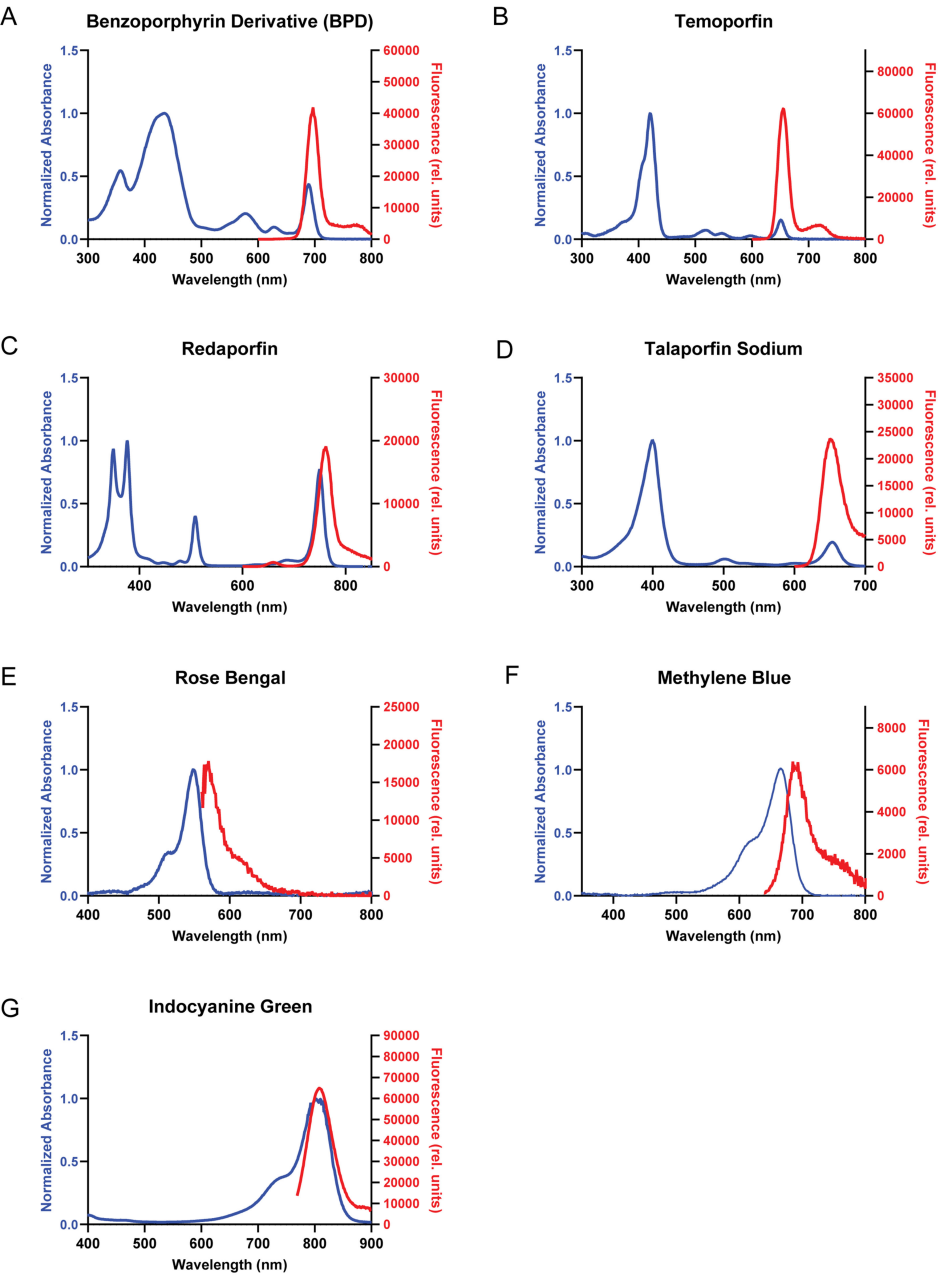
Normalized absorbance (blue) and fluorescence (red) spectra of (A) BPD, (B) temoporfin, (C) redaporfin, (D) talaporfin sodium, (E) rose bengal, (F) methylene blue and (G) ICG used in the study. The absorbance spectrum of the test photosensitizers was recorded using a spectrophotometer. The photosensitizers were excited at respective Soret and Q bands to record the emission spectrum of the photosensitizers. BPD Ex: 435 nm; Temoporfin Ex: 422 nm; Redaporfin Ex: 510 nm; Talaporfin sodium Ex: 398 nm; Rose bengal Ex: 520 nm; Methylene blue Ex: 610 nm; ICG Ex: 730 nm. Respective excitation wavelengths at the Soret or Q band were chosen to avoid overlap between the excitation and the emission wavelengths while recording the fluorescence intensity during extraction experiments. BPD: Benzoporphyrin derivative; ICG: indocyanine green.

To evaluate the effect of the ABC transporter on the photosensitizer concentration in the cells, uptake studies were conducted using a panel of MCF-7 sublines that overexpressed ABCG2, P-gp, and MRP1. The intracellular fluorescence of photosensitizers was measured at specific excitation and emission wavelengths [Figure 3]. Intracellular photosensitizer accumulation for experimental groups (NT, PS, and PS+I) was quantified using fluorescence intensity standard curves [Supplementary Figure 1].

**Figure 4:**
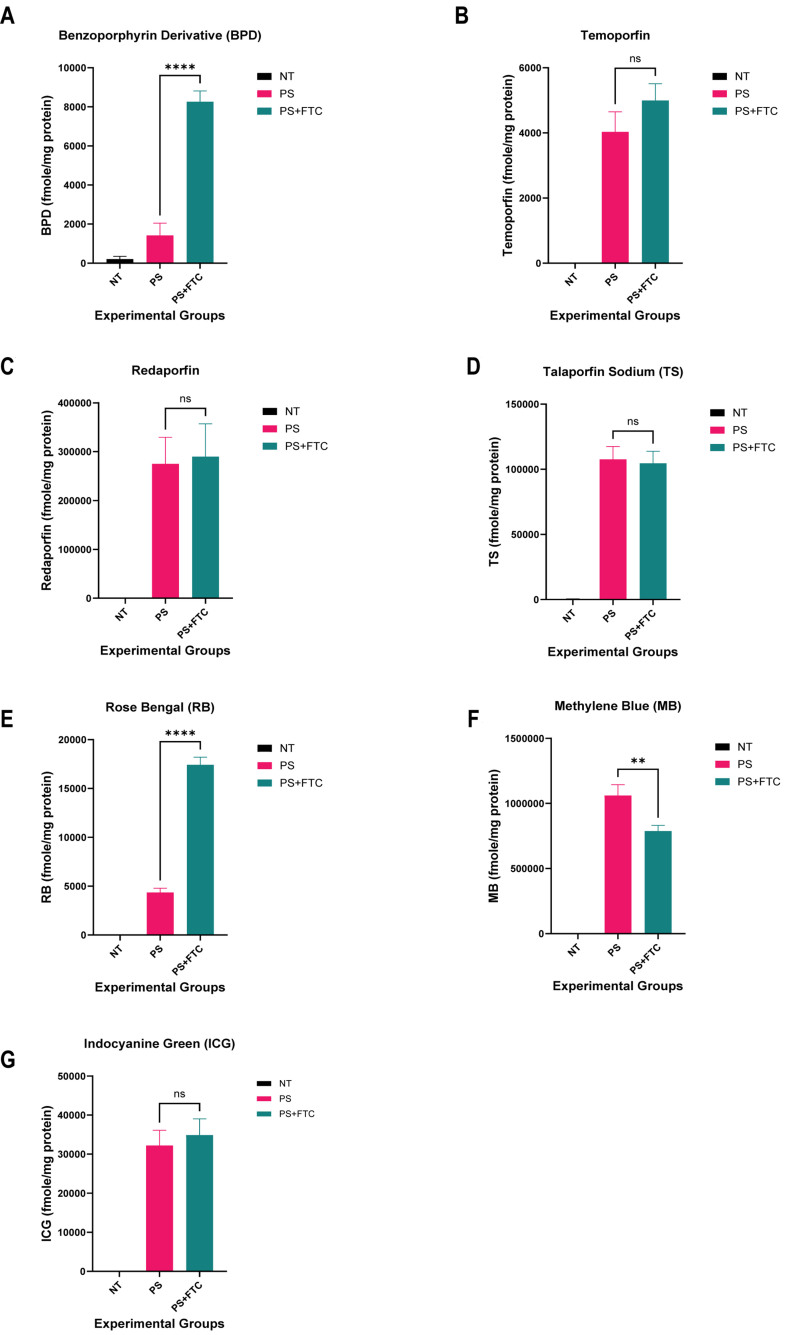
Intracellular levels of photosensitizers in MCF-7 MX100 (ABCG2-overexpressing) cell line using extraction method. Intracellular levels of the photosensitizers from cell lysates were determined by the fluorescence signals of the photosensitizer. BPD and rose bengal show a significant increase in accumulation in MCF-7 MX100 cells in the presence of ABCG2 inhibitor (FTC). The fmole/mg protein of photosensitizer is plotted for all the experimental groups. (A) Benzoporphyrin derivative; (B) Temoporfin; (C) Redaporfin; (D) Talaporfin sodium; (E) Rose Bengal; (F) Methylene blue; (G) Indocyanine green. (*P < 0.05, **P < 0.01, ***P < 0.001; One-way ANOVA Tukey’s range test) (*N* = 3-6). NT: not treated control; PS: photosensitizer only; PS+FTC: photosensitizer + inhibitor; ns: non-significant; BPD: benzoporphyrin derivative; FTC: fumitremorgin C.

In ABCG2-overexpressing cells [Figure 4], ABCG2 inhibitor FTC notably augmented the intracellular accumulation of BPD [Figure 4A] and rose bengal [Figure 4E]. However, no significant difference was observed in the intracellular fluorescence of temoporfin, talaporfin sodium, redaporfin, and ICG [Figure 4B-D and F-G]. The intracellular accumulation of methylene blue decreased significantly in the presence of FTC, suggesting competitive inhibition between methylene blue and FTC. Nevertheless, incubation with Ko143, a known ABCG2 inhibitor, showed no change in intracellular methylene blue levels between the PS and PS+I groups, suggesting no interaction between methylene blue and ABCG2 [Supplementary Figure 2]. The results suggest that BPD and rose bengal are substrates of the ABCG2 transporter.

**Figure 5:**
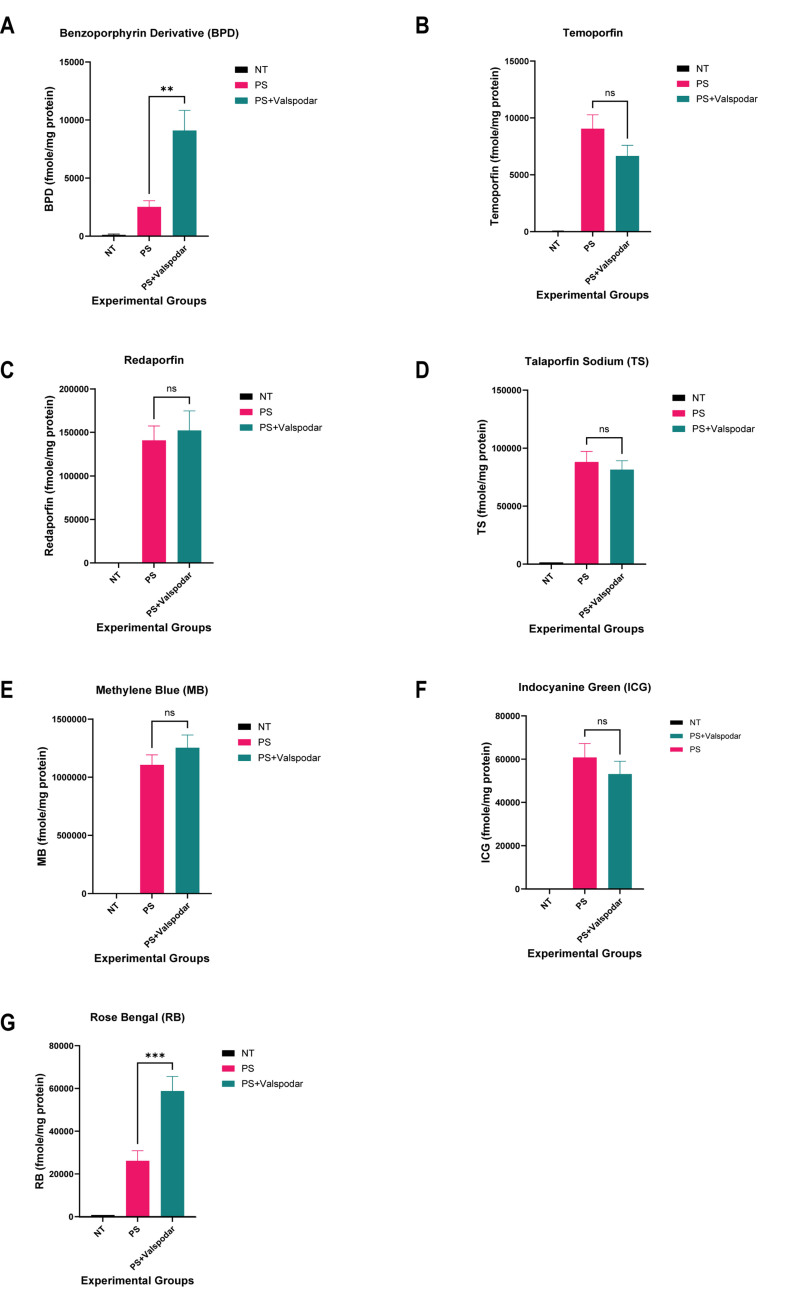
Intracellular levels of photosensitizers in MCF-7 TX400 (P-gp overexpressing) cell line using extraction method. Intracellular levels of the photosensitizers from cell lysates were determined by the fluorescence signals of the photosensitizer. BPD and rose bengal show a significant increase in accumulation in MCF-7 TX400 cells in the presence of a P-gp inhibitor (valspodar). The fmole/mg protein of the photosensitizer was plotted for all the experimental groups. (A) Benzoporhyrin derivative; (B) Temoporfin; (C) Redaporfin; (D) Talaporfin sodium; (E) Methylene blue; (F) Indocyanine green; (G) Rose bengal. *P < 0.05, **P < 0.01, ***P < 0.001; One-way ANOVA Tukey’s range test (*N* = 3-5). NT: Not treated, control; PS: photosensitizer only; PS+Valspodar: photosensitizer + inhibitor; ns: non-significant; BPD: benzoporphyrin derivative.

In P-gp overexpressing MCF-7 TX400 cells, adding the P-gp inhibitor significantly increased the intracellular accumulation of BPD compared to the respective PS group [Figure 5A]. No significant increase in photosensitizer accumulation was observed for temoporfin, redaporfin, talaporfin sodium, methylene blue, and ICG [Figure 5B-F]. The intracellular accumulation of rose bengal showed a significant difference compared to the PS group [Figure 5G]. The results obtained with extraction studies with MCF-7 TX400 cells suggest the potential interaction of BPD and rose bengal with the P-gp transporter.

**Figure 6:**
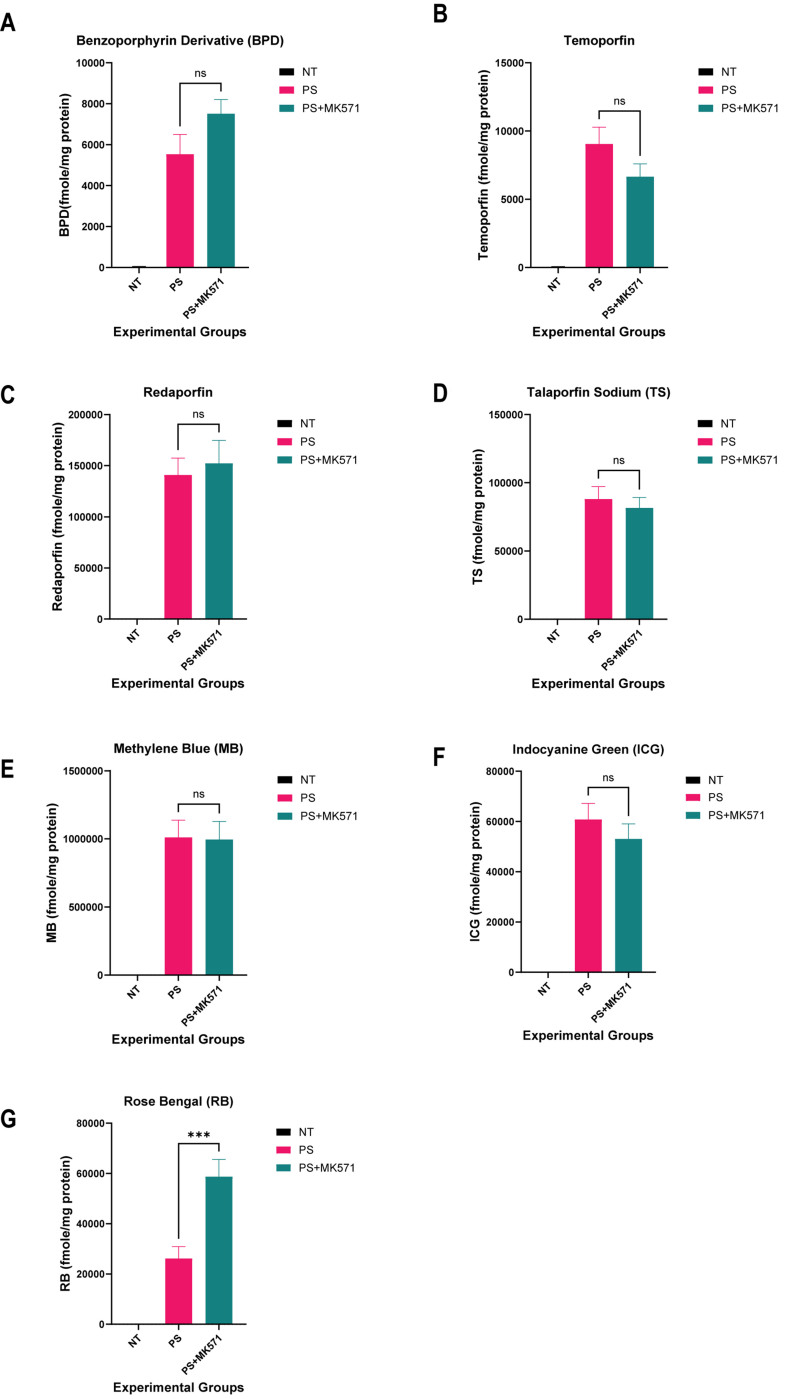
Intracellular levels of photosensitizers in MRP1-overexpressing MCF-7/VP cell line using extraction method. Intracellular levels of the photosensitizers from cell lysates were determined by the fluorescence signals of the photosensitizer. Rose bengal shows a significant increase in accumulation in MCF-7/VP cells in the presence of MRP1 inhibitor (MK571). The fmole/mg protein of photosensitizer is plotted for all the experimental groups. (A) Benzoporhyrin derivative; (B) Temoporfin; (C) Redaporfin; (D) Talaporfin sodium; (E) Methylene blue; (F) Indocyanine green; (G) Rose bengal. *P < 0.05, **P < 0.01, ***P < 0.001; One-way ANOVA Tukey’s range test (*N* = 3-5). NT: Not treated control; PS: photosensitizer only; PS+MK571: photosensitizer + inhibitor; ns: non-significant.

For the MRP1-overexpressing cell line, the addition of MK571 (MRP1 inhibitor) led to no significant photosensitizer accumulation in the presence and absence of MK571 for BPD and temoporfin [Figure 6A and B]. The results obtained for redaporfin, talaporfin sodium, methylene blue, and ICG showed no significant photosensitizer accumulation in the presence of the MK571 inhibitor compared to the group without the inhibitor [Figure 6C-F]. A significant accumulation of rose bengal was observed in the PS+I group compared to the PS group [Figure 6G]. These results suggest that only BPD and rose bengal are substrates of ACBG2 and P-gp.

### ABC transporter-mediated effects on photosensitizer accumulation in MCF-7 breast cancer sublines investigated by flow cytometry

**Figure 7:**
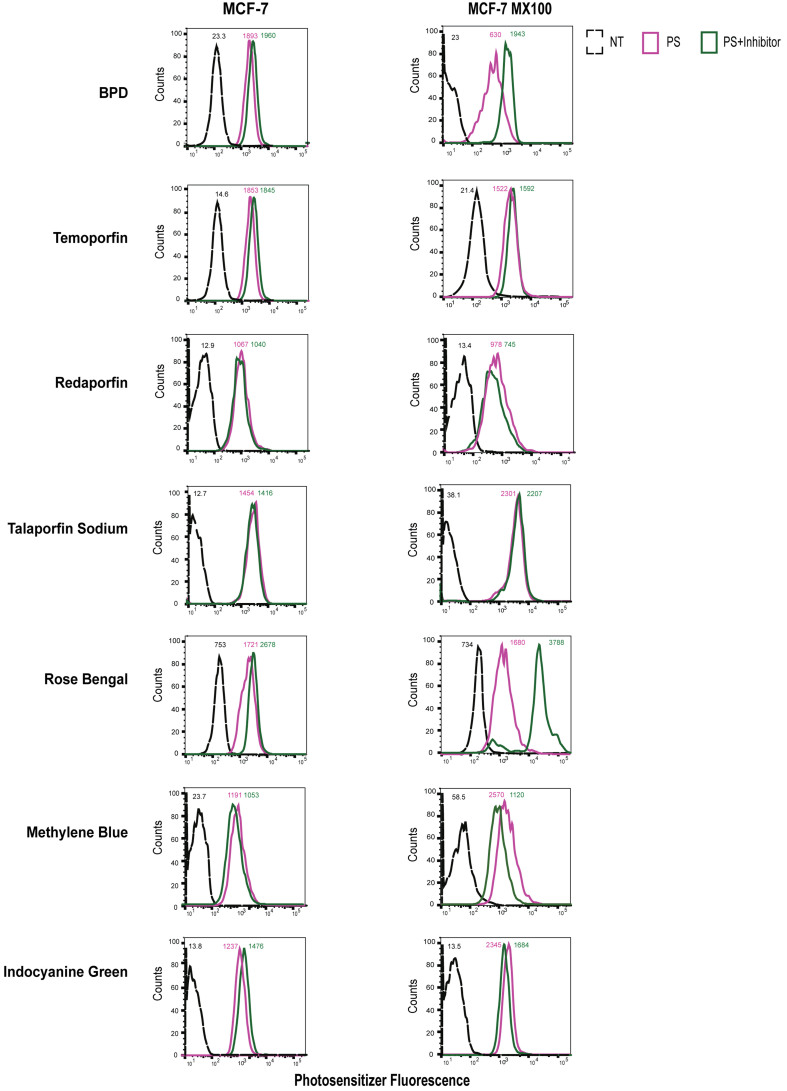
ABCG2-overexpressing cells transport selected photosensitizers (BPD and Rose Bengal). Flow cytometry was performed to assess the intracellular fluorescence of photosensitizer. The raw mean fluorescence intensity values are reported for individual histograms. BPD and Rose bengal show higher uptake in the presence of the ABCG2 inhibitor, FTC. NT: Not treated, control; PS: photosensitizer only; PS+Inhibitor: photosensitizer + FTC; BPD: benzoporphyrin derivative; FTC: fumitremorgin C.

Flow cytometry was performed to confirm the results obtained with the extraction method approach and assess the effect of ABC transporters on photosensitizer accumulation. The fluorescence signal of photosensitizers was recorded using flow cytometry. In MCF-7 MX100 cells, the addition of FTC resulted in increased fluorescence of both BPD and rose bengal [Figure 7]. MCF-7 MX100 cells were also found to accumulate less methylene blue than the PS group when incubated with the FTC inhibitor. Notably, FTC led to a slight decrease in intracellular methylene blue fluorescence in the parental cell line, indicating limited transportation of methylene blue by ABCG2. A similar trend was also observed with the extraction studies performed with methylene blue and FTC. Intracellular levels of temoporfin, redaporfin, talaporfin sodium, and ICG remained largely unaffected by the presence of FTC in both MCF-7 MX100 and parental cells [Figure 7].

**Figure 8:**
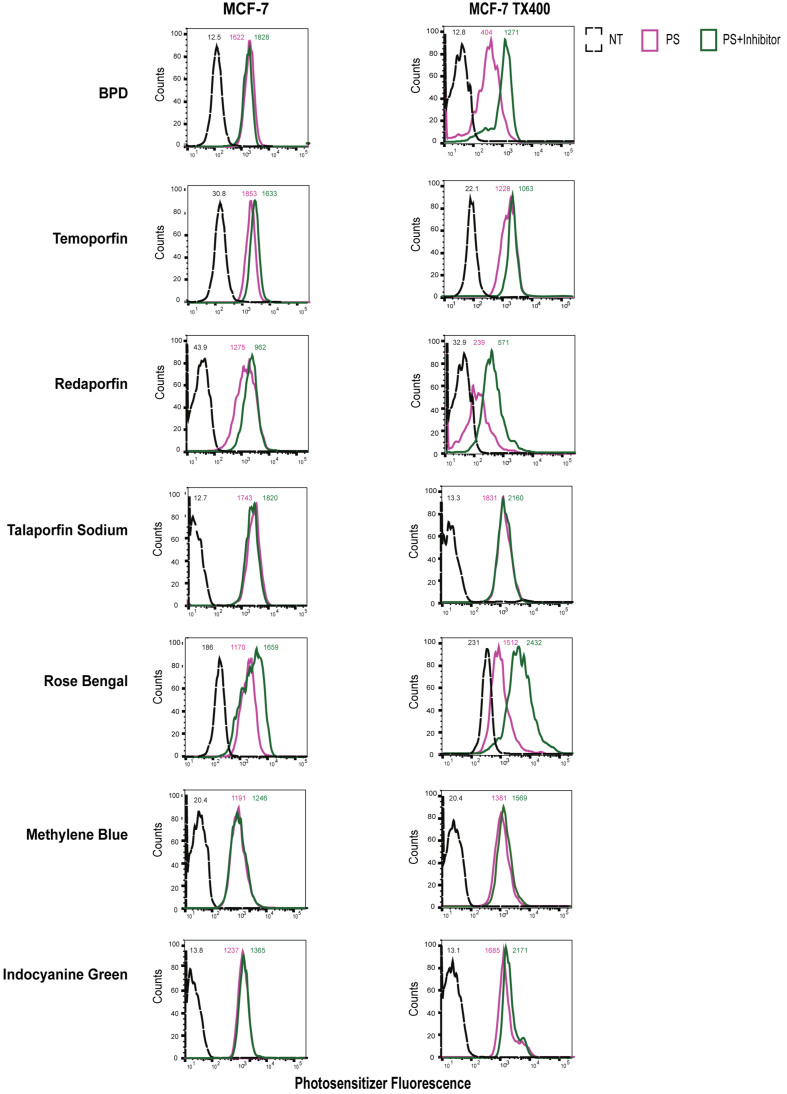
P-gp-overexpressing cells transport selected photosensitizers (BPD, Rose Bengal, and Redaporfin*). Flow cytometry was performed to assess the intracellular fluorescence of photosensitizer. The raw mean fluorescence intensity values are reported for individual histograms. BPD, redaporfin, and rose bengal show higher uptake in the presence of P-gp inhibitor valspodar. Heterogeneity in the single-cell population for the redaporfin-only group was observed in MCF-7 TX400 cells. NT: Not treated, control; PS: photosensitizer only; PS+Inhibitor: photosensitizer + valspodar; BPD: benzoporphyrin derivative.

In Figure 8, upon treatment of P-gp-overexpressing with 2 μM of BPD, a noticeable decrease in fluorescence intensity was observed in the PS group. However, when cells were treated with BPD and valspodar, a notable increase in BPD fluorescence was observed in TX400 cells. An increase in rose bengal intracellular fluorescence was also observed in the PS+I group compared to the PS group. However, this trend was not evident in the parental cell line. Thus, elevated levels of valspodar-inhibitable efflux of both BPD and rose bengal were observed. Valspodar also increased the intracellular fluorescence intensity of redaporfin in P-gp-overexpressing cells. The intracellular fluorescence intensities of temoporfin, talaporfin sodium, methylene blue, and ICG remained largely unchanged when MCF-7 TX400 and parental cells were treated with photosensitizers in the presence of valspodar.

**Figure 9:**
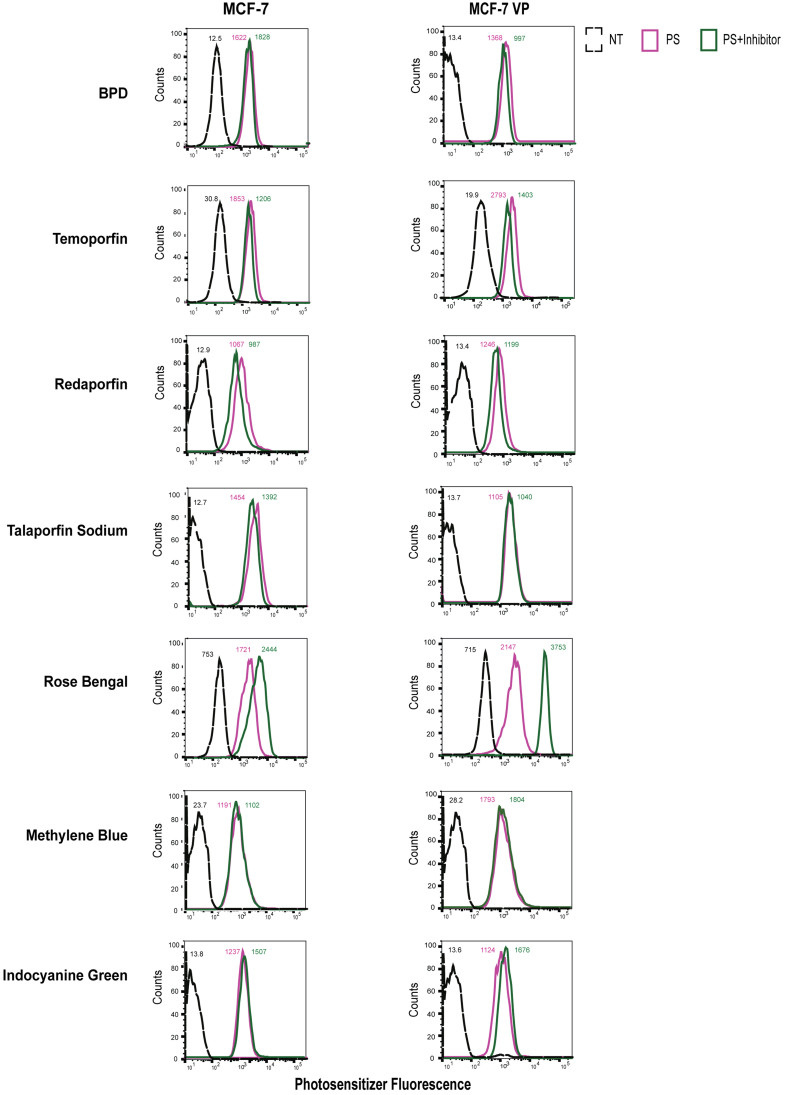
MRP1 transports a selected photosensitizer (Rose Bengal). Flow cytometry was performed to assess the intracellular fluorescence of photosensitizer. The raw mean fluorescence intensity values are reported for individual histograms. Rose bengal shows higher uptake in the presence of MRP1 inhibitor MK571. NT: Not treated, control; PS: photosensitizer only; PS+Inhibitor: photosensitizer + MK571.

MCF-7/VP cells demonstrated appreciable MRP1-mediated transport of rose bengal, as demonstrated by the PS (pink line) and PS+I group (green line) [Figure 9]. However, only a slight increase in the P+I group was observed in the MCF-7 parental cells. MRP1 transported none of the tested photosensitizers besides rose bengal (Figure 9, column 2).

These results indicate that ABCG2 and P-gp translocate BPD but not MRP1 as expected. Rose bengal, on the other hand, is a substrate for ABCG2, P-gp, and MRP1 transporter. However, none of the other tested photosensitizers showed ABCG2, P-gp, and MRP1-mediated transport in MCF-7 cell lines overexpressing ABC transporters. The normalized flow cytometry fluorescence intensities are reported in Supplementary Table 3.

### ABC transporter-photosensitizer interactions affect PDT-mediated phototoxicity

**Figure 10:**
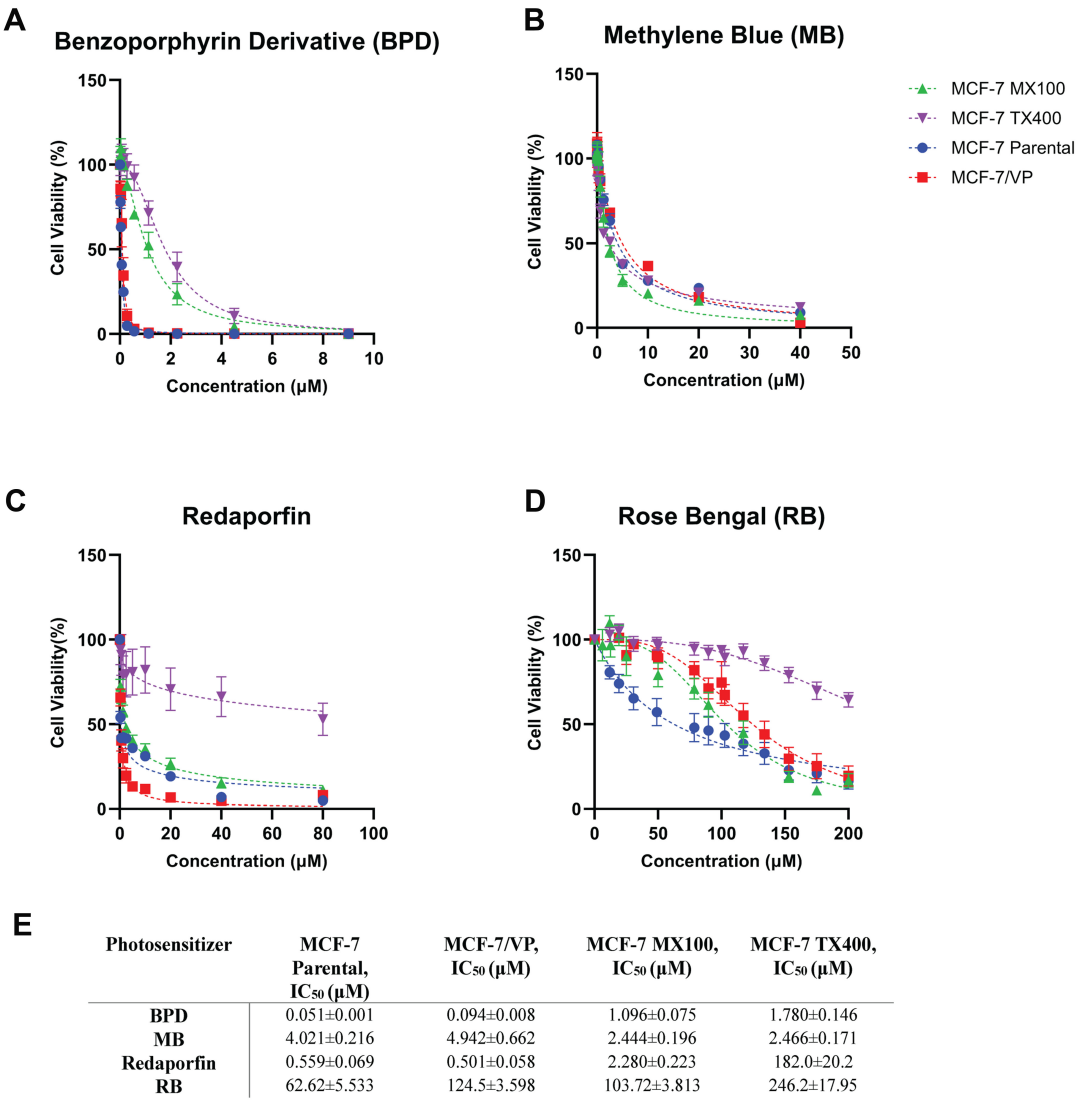
Photosensitizers that are ABC transporter substrates are resistant to PDT. Cell viability assays were performed 24 h post-PDT for (A) BPD, (B) Methylene Blue, (C) Redaporfin, and (D) Rose Bengal; (E) The IC_50_ (reported in μM) for each photosensitizer for the respective cell line is summarized in the table. ABC: ATP binding cassette; PDT: photodynamic therapy; BPD: benzoporphyrin derivative.

To assess whether ABC transporter-mediated translocation of photosensitizers observed with extraction and flowcytometry studies for BPD, rose bengal, and redaporfin would result in resistance from PDT, cell viability assays were performed using MCF-7 sublines and parental cells [Figure 10]. The IC_50_ values obtained for respective cell lines are summarized in Figure 10E. In agreement with the results obtained by flow cytometry, redaporfin showed resistance to PDT in MCF-7 TX400 cells overexpressing P-gp. The IC_50_ of redaporfin in MCF-7 TX400 cells (182 20.2 M) was determined to be significantly higher than the IC_50_ of redaporfin in parental cells (0.559 0.069 M). No significant difference in IC_50_ was observed in ABCG2 and MRP1 cells compared to parental cells for redaporfin.

In contrast, methylene blue did not show significant PDT resistance in any of the MCF-7 sublines overexpressing ABCG2, P-gp, or MRP1. The IC_50_ values for methylene blue were similar between the parental cells and the ABC transporter-overexpressing sublines. However, PDT resistance was observed in all MCF-7 sublines when treated with rose bengal. A significant increase in IC_50_ concentrations was observed for rose bengal in MCF-7 MX100, MCF-7 TX400, and MCF-7/VP cells compared to the parental cells.

## DISCUSSION

Drug resistance continues to be a leading cause of treatment failure and remains a significant challenge in cancer therapies. Multidrug resistance (MDR) involves various intrinsic and acquired factors that help evade the cytotoxic effects of structurally and functionally unrelated drugs^[43]^. Previous studies have demonstrated a positive correlation between ABC transporter co-expression and decreased relapse-free survival in cancer patients^[44-46]^. PDT is a promising approach to address drug resistance in cancer^[47]^. Various studies have examined the effectiveness of PDT in overcoming MDR and resensitizing tumor cells to treatment. For instance, previous studies have shown the efficacy of temoporfin-mediated PDT against 5-fluorouracil-resistant cancer cells^[48]^. A previous study with BPD demonstrated the downregulation of ABCG2 expression at a low PDT dose with an improved uptake of irinotecan in human pancreatic cancer cells. Another study reported a decrease in the uptake of PhA photosensitizer in HT-29 human colorectal adenocarcinoma cells overexpressing ABCG2 both *in vivo* and *in vitro*. The treatment efficacy improved in the presence of Ko143 (ABCG2 inhibitor)^[49]^. Since effective PDT relies on the intracellular localization of photosensitizers, the crucial question arises regarding whether these photosensitizers are substrates of ABC drug efflux transporters responsible for PDT resistance in cancer cells^[50]^.

Given the limited data on the impact of ABC drug transporters on photosensitizer efflux, the ability of ABCG2, P-gp, and MRP1 to decrease the intracellular accumulation was determined for a panel of clinically relevant photosensitizers: BPD, temoporfin, redaporfin, talaporfin sodium, rose bengal, methylene blue, and ICG using both quantitative (extraction) and qualitative (flow cytometry) methods. The results obtained with the optimized extraction method for BPD (known substrate for ACBG2 and P-gp, but not MRP1) and temoporfin (not a substrate of ABCG2, P-gp, and MRP1) were consistent with previous findings^[36,41]^. No significant increase in fluorescence intensity of temoporfin was observed in the panel of ABC transporter-overexpressing cells in the presence of respective inhibitors measured using flow cytometry. BPD, as expected, exhibited increased intracellular fluorescence intensity in ABCG2- and P-gp-overexpressing cells in the presence of FTC and valspodar inhibitors, respectively, demonstrating efflux of BPD through these transporters.

In the presence of FTC inhibitor, cells overexpressing ABCG2 also showed increased fluorescence intensity of rose bengal compared to parental cells. While our observations are in agreement with previous findings^[36,41]^, this study provides new knowledge that redaporfin, talaporfin sodium, and ICG are not substrates of ABCG2. However, the intracellular levels of methylene blue decreased in ABCG2-overexpressing cells in the presence of FTC inhibitor. A similar trend was also observed while measuring the fluorescence intensity of methylene blue in MCF-7 MX100 cells in the presence of FTC with flow cytometry. FTC is the first and widely known ABCG2 inhibitor to modulate drug resistance in cancer therapies. However, FTC is precluded from clinical use due to off-target effects that lead to undesirable neurotoxicity^[51,52]^. The significant decrease in methylene blue accumulation in the presence of FTC compared to the methylene blue-only group suggests potential off-target interactions with other cellular components that might affect MB uptake, thereby reducing methylene blue uptake in the presence of FTC. On the other hand, extraction experiments with a more potent and specific ABCG2 inhibitor, Ko143, showed no significant difference in methylene blue accumulation between methylene blue-only and methylene blue+Ko143 groups [Supplementary Figure 2].

The interaction of the tested panel of photosensitizers with P-gp and MRP1 transporters was also explored. None of the photosensitizers except rose bengal were found to be substrates of P-gp. Our results with flow cytometry data for redaporfin show a five-fold increase in fluorescence intensity with valspodar in P-gp-overexpressing cells. However, the extraction results did not show this trend [Figure 5C]. Additionally, the broader histogram of fluorescence intensity distribution for the PS groups for MCF-7 TX400 cells indicates a heterogeneous drug uptake profile within the cell population.

In addition to PDT, photosensitizers are also being explored as photodiagnostic agents for fluorescence-guided interventions like fluorescence-guided surgery (FGS) applications in the clinic^[53]^. Table 1 summarizes the ABC transporter substrate status of clinically relevant photosensitizers. Our results indicate that in tumors expressing P-gp, ABCG2, and MRP1, fluorescence imaging or PDT of tumors could potentially be less effective with photosensitizers that are substrates of ABC transporters.

**Table 1:** ABC transporter substrate status of clinically relevant photosensitizers

**Photosensitizers**	**Substrate of ACBG2**	**Substrate of P-gp**	**Substrate of MRP1**	**Ref.**
Rose bengal	Yes	Yes	Yes	
BPD	Yes	Yes	No	[36,54]
Temoporfin/Foscan®	No	No	No	[41,55]
Methylene blue	No	No	No	
Redaporfin	No	Yes	No	
Talaporfin sodium	No	No	No	
Indocyanine green	No	No	No	
5-ALA/PpIX	Yes	No	No	[41,56]
Chlorin-e6	Yes	No	No	[40,41]
Pheorphorbide a	Yes	No	No	[38,39,41]
MPPa	Yes	No	No	[41]
HpD	No	No	No	[41]

ABC: ATP binding cassette; BPD: benzoporphyrin derivative; MPPa: pyropheophorbide, a methyl ester; HpD: hematoporphyrin.

Although the results from the *in vitro* assays from the study confirm the impact of ABC transporter inhibition on the accumulation and efflux of the photosensitizers, further investigations employing molecular docking analysis are warranted to understand potential interactions between rose bengal, redaporfin, and the substrate binding sites of ABCG2, P-gp, and MRP1. Given the incomplete understanding of transporter substrate interaction, employing computational models to predict interaction probabilities of photosensitizers with ABC efflux transporters would provide insights that can inform the development of effective PDT strategies for MDR in cancer patients^[57-59]^.

Thus, photosensitizers that are substrates of ABC transporters may be less effective in treating cancers expressing these transporters. We have also observed that resistance to redaporfin-mediated photodynamic therapy (PDT) increased in cells expressing P-gp, as indicated by a significantly higher IC_50_ compared to parental cells [Figure 10]. This observation was consistent with flow cytometry results and suggests that P-gp translocates redaporfin. Additionally, PDT resistance was noted with Rose Bengal in cells overexpressing ABCG2, P-gp, and MRP1. Conversely, BPD exhibited a marked increase in IC_50_ in MCF-7 MX100 and MCF-7 TX400 cells, aligning with its known status as a substrate for ABCG2 and P-gp but not MRP1. Cells overexpressing ABC transporters did not show resistance to PDT with Methylene Blue. This limitation of PDT resistance, however, can be overcome by leveraging the potential synergy between photosensitizers and ABC transporter inhibitors to overcome drug resistance and enhance the efficacy of PDT in cancer treatment^[60,61]^. While *in vitro* studies have shown enhanced chemosensitivity with inhibitors, none of the agents have been FDA-approved for the modulation of ABC transporters to overcome drug resistance in cancer patients. Clinical trials with various generations of ABC transporter inhibitors showed limited benefits to cancer patients due to inherent toxicity (first-generation ABC inhibitors), off-target effects, potential drug-drug interactions (second-generation ABC inhibitors), and narrow therapeutic range for targeted transporter inhibition (third-generation ABC inhibitors)^[57,62]^. In addition, other mechanisms like tumor heterogeneity and co-expression of ABC transporters ABCG2, P-gp, and MRP1 with overlapping substrate specificities pose further challenges to overcome MDR. Thus, the selective inhibition of one ABC transporter can be compensated by the expression of other efflux transporters, causing limited treatment efficacy^[63]^. Furthermore, drug repurposing strategies using clinically approved agents, such as tyrosine kinase inhibitors, are being explored to resensitize drug-resistant cancer cells to chemotherapy^[57,64-66]^.

Alternatively, our previous findings have shown that light activation of photosensitizers-substrates could affect ABC transporter protein expression, ATPase activity, and intracellular mitochondrial ATP levels *in vitro* and improve irinotecan drug accumulation *in vitro* and in tumor tissues^[42,59,67]^. Further studies are needed to confirm the PDT efficacy of photosensitizers that are not substrates of ABC transporters, which can escape efflux by these transporters and circumvent drug resistance.

It is important to note that MDR is a complex phenomenon caused by multiple mechanisms that build a complex network of molecular mechanisms mediating the MDR phenotype. Despite temoporfin and talaporfin sodium not being a substrate of ABC transporters, resistance to PDT with the agents in cancer cells has been observed, possibly due to mechanisms such as loss of p53 function and Ras expression^[68-71]^. Thus, a comprehensive understanding of drug resistance mechanisms is needed to design effective treatment strategies for cancer.

In summary, we have shown that photosensitizers BPD and rose bengal are transported by ABCG2 and P-gp, while ABCG2 does not efflux redaporfin, temoporfin, talaporfin sodium, methylene blue, and ICG. Our results also suggest that rose bengal is also transported by MRP1. This study also provides new knowledge that ABCG2, P-gp, and MRP1 transporters do not affect the intracellular accumulation of redaporfin (potential interactions with P-gp), talaporfin sodium, methylene blue, and ICG photosensitizers. Additional research is needed to investigate the combination effect of PDT with repurposed ABC transporter inhibitors for safe clinical translation.

## DECLARATIONS

### Availability of data and materials

Data available within the article and the Supplementary Materials.

### Copyright

© The Author(s) 2024.
