# Supplementary material for: Screening of photosensitizers-ATP binding cassette (ABC) transporter interactions in vitro
Source: Cancer Drug Resist. 2024 Sep 21;7:35. doi: 10.20517/cdr.2024.50 (PMC11472579; doi:10.20517/cdr.2024.50)
Supplement: Supplementary file 1 [file cdr-7-35-SupplementaryMaterials.pdf]

Vig *et al. Cancer Drug Resist* 2024;7:35
DOI: 10.20517/cdr.2024.50

Cancer Drug Resistance

Supplementary Materials

Screening of photosensitizers-ATP binding cassette (ABC) transporter interactions *in-vitro*

Shruti Vig[1], Payal Srivastava[1*], Idrisa Rahman[1,2*], Renee Jaranson[1], Anika Dasgupta[1], Robert Perttilä[3], Petteri Uusimaa[3], Huang Chiao Huang[1#]

[1]*Fischell Department of Bioengineering, University of Maryland, College Park, MD 20742, USA*

[2]*Laboratory of Cell Biology, Center for Cancer Research, National Cancer Institute, National Institutes of Health, Bethesda, MD 20892, USA*

[3]*Modulight Corporation, Tampere FI-33720, Finland*

**Equal contribution*

#Corresponding Author; Huang-Chiao Huang (hchuang@umd.edu)

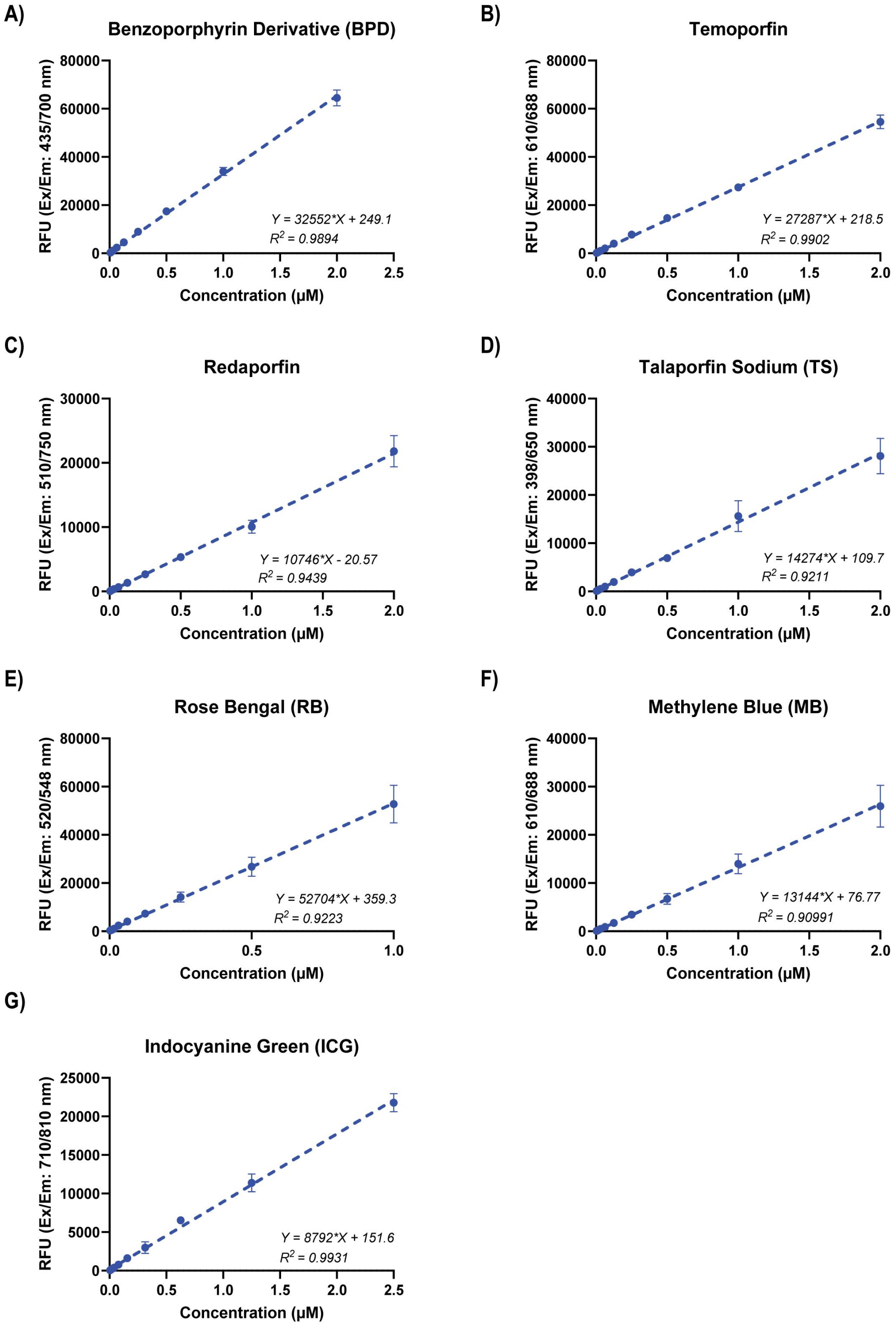

Supplementary Figure 1. Fluorescence intensity standard curves for tested photosensitizers. The tested photosensizers were excited at specific excitation and emission wavelengeths photosensitizer [BPD: Ex/Em: 430/700; ICG: 710/810 nm; MB: Ex/Em: 610/688 nm; RB: Ex/Em: 520/575; Temoporfin: Ex/Em 422/660 nm: 430/700; Redaporfin: Ex/Em: 510/750 nm; and Talaporfin sodium: Ex/Em: 398/650 nm]. The figure plots show the mean± standard error fluorescence intensity obtained at known concetration of the photosensitizer. The unknown fluorescence intensity for photosensitzer obtained from cell lysates was extrapolated using the standard curve linear equation to obtain the intracellular concentration of the photosensitzer. The standard curves show a consistent linear trend at the plotted concentrations.

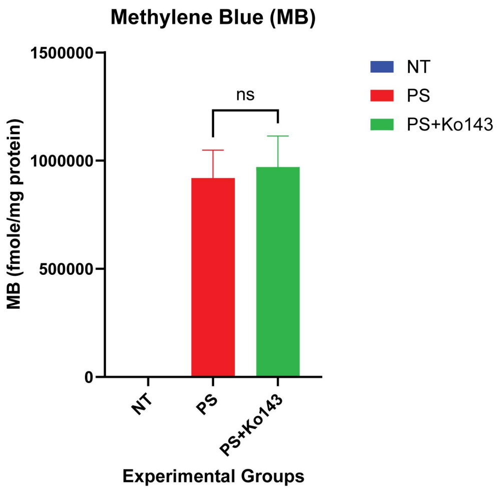

Supplementary Figure 2. Intracellular accumulation of Methylene Blue in MCF-7 MX100 cells (ABCG2) in presence of ABCG2 inhibitor Ko143. The intracellular accumulation of Methylene Blue was quantified using the extraction method stated in the method section using Ko143 inhibitor. No significant difference in intracellular accumulation of Methylene Blue was observed between the PS and PS+Ko143 group.

Supplementary Table 1. Summary of selected clinically used photosensitizers and their applications

Name	Alternative names	MW (g/mol)	Structure and chemical formula	Approval	Status of use
Benzoporphyrin Derivative (BPD)	Verteporfin, Visudyne®, BPD-MA	718.8	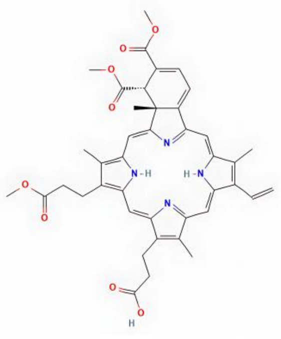<br>$C_{41}H_{42}N_4O_8$[1]	Approved by the FDA in 2000 for sub foveal choroidal neovascularization due to age related macular degeneration (AMD), pathologic myopia, or presumed ocular histoplasmosis	NCT04429139, NCT03033225, NCT06306638, NCT02872064, NCT02464761, NCT00049959, NCT02702700, NCT03067051, NCT02939274, NCT00002647, NCT06381154, NCT00007969)
Temoporfin	Foscan, meso-Tetrahydroxyphenylchlorin, m-THPC, Foslip, 5,10,15,20-tetra(m-hydroxyphenyl)chlorin	680.7	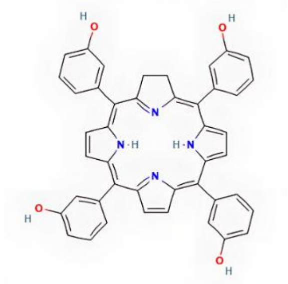<br>$C_{44}H_{32}N_4O_4$[2]	Approved by European Medical Agency (EMA) for advanced head and neck cancer in 2001, Phase I/II clinical trials in the US for bile duct carcinoma and lung cancer	NCT03003065, NCT01854684, NCT01415986, NCT01637376, NCT01718223, NCT01086488, NCT00003856
Redaporfin	LUZ11, 5,10,15,20-tetrakis(2,6-difluoro-3-N-methylsulfamoylphenyl)bacteriochlorin	1,135.1	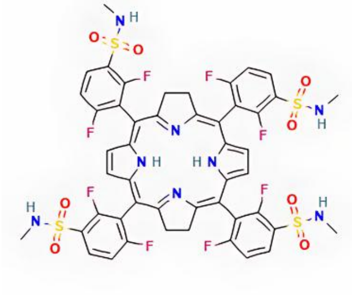<br>$C_{48}H_{38}F_8N_8O_8S_4$[3]	Received an orphan drug designation from the EMA in 2016, Phase I/II trials for head and neck cancer	NCT02070432
Talaporfin Sodium	Laserphyrin, NPe6, LS11, mono-L-aspartyl chlorin e6	799.7	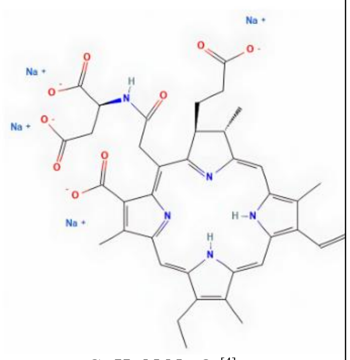<br>$C_{38}H_{37}N_5Na_4O_9$[4]	Approved in Japan in 2004 for early stage lung cancer, Phase III trials for hepatocellular carcinoma, Phase II trials for refractory colorectal liver metastases	NCT00122876, NCT00716469, NCT00068068, NCT00083785, NCT00028405)

Rose Bengal	Robengatope (brand name), Rose Bengal sodium I-131, PV-10 sodium	1,017.6	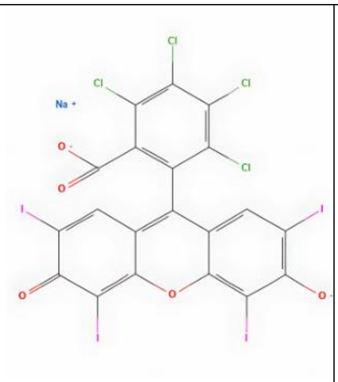<br>$C_{20}H_2Cl_4I_4Na_2O_5$[5]	Phase I/II studies for treatment of neuroendocrine tumors and melanoma	NCT02693067, NCT01760499, NCT00237354, NCT00521053
Methylene Blue	Methylthioninium chloride, basic blue-9, urolene blue, provayblue	319.9	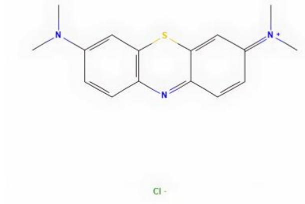<br>$C_{16}H_{18}ClN_3S$[6]	Approved by the FDA for the treatment of pediatric and adult patients with acquired methemoglobinemia	NCT00784849, NCT01520324, NCT04842968, NCT02982148
Indocyanine Green	ICG, IC-Green, cardiogreen, foxgreen, (Green, Indocyanine), Ujoveridin, Vofaverdin, Vophaverdin, Wofaverdin, Cardio-Green, Cardio Green, Cardiogreen	775.0	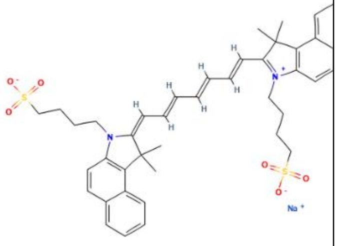<br>$C_{43}H_{47}N_2NaO_6S_2$[7]	FDA approved for cardiac output, hepatic function and liver blood flow, and for ophthalmic angiography	NCT0125375

Supplementary Table 2. The molar extinction coefficients(MEC) of the panel of photosenstizers

Photosensitizer	MEC (@wavelength)	Solvent	Ref.
Benziporphyrin derivative	34,895 $M^{-1} \cdot cm^{-1}$ (687 nm)	DMSO	[8]
Temoporfin	26,776 ± 1,085.51 $M^{-1} \cdot cm^{-1}$ (651 nm)	DMSO	Calculated
Redaporfin	67,537.3 ± 2,771.2 $M^{-1} \cdot cm^{-1}$ (743 nm)	DMSO	Calculated
Talaporfin sodium	23,458 $M^{-1} \cdot cm^{-1}$ (651 nm)	PBS	Calculated
Rose bengal	90,400 $M^{-1} \cdot cm^{-1}$ (559 nm)	Water	[9]
Methylene blue	7.41×10^4 $M^{-1} \cdot cm^{-1}$ (665 nm)	Water	[10]
Indocyanine Green (ICG)	262,100 $M^{-1} \cdot cm^{-1}$ (780 nm)	Water	[11]

The table shows the MEC values of the panel of photosensitzers tested in this study. The molar extinction coefficient of photosensitzers were calculated for Temoporfin, Redaporfin and Talaporfin sodium and specifc wavelengths. The MEC for BPD, RB, MB and ICG was obtained from the literature.

Supplementary Table 3. Normalized fluorescence intensity of panel of tested photosensitizers

Photosensitizers	ABCG2	ABCG2+FTC	P-gp	P-gp+Valspodar	MRP1	MRP-1+MK571
Rose bengal	0.78 ± 0.18	2.42 ± 0.19	0.93 ± 0.32	1.48 ± 0.52	1.52 ± 0.41	4.45 ± 0.03
Benzoporhyrin derivative (BPD)	0.45 ± 0.17	1.00 ± 0.06	0.22 ± 0.03	0.86 ± 0.07	1.15 ± 0.31	0.78 ± 0.24
Temoporfin/Foscan®	0.96 ± 0.20	1.03 ± 0.24	0.79 ± 0.17	0.55 ± 0.04	1.37 ± 0.20	0.70 ± 0.08
Methylene blue	1.42 ± 0.64	1.04 ± 0.11	0.93 ± 0.25	0.98 ± 0.32	1.16 ± 0.31	1.22 ± 0.28
Redaporfin	0.89 ± 0.42	0.82 ± 0.49	0.20 ± 0.05	**0.57 ± 0.18***	0.83 ± 0.30	0.73 ± 0.34
Talaporfin sodium	1.59 ± 0.01	1.59 ± 0.11	1.03 ± 0.04	1.20 ± 0.13	0.97 ± 0.30	0.84 ± 0.18
Indocyanine green	1.28 ± 0.44	1.68 ± 0.54	0.72 ± 0.68	0.99 ± 0.90	0.89 ± 0.11	1.14 ± 0.20

The table hows the average normalized fluorescence intensity values for tested agents using flow cytometry. The fluorescence intensity was normalized to signal from the control parental cell line.

REFERENCES

1. Information, N.C.f.B. PubChem Compound Summary for CID 5362420, Visudyne. [cited 2024 July 4]; Available from: https://pubchem.ncbi.nlm.nih.gov/compound/Visudyne.
2. Information, N.C.f.B. PubChem Compound Summary for CID 60751, Temoporfin. [cited 2024 July 4]; Available from: https://pubchem.ncbi.nlm.nih.gov/compound/Temoporfin.
3. Information, N.C.f.B. ubChem Compound Summary for CID 86287614, Redaporfin. [cited 2024 July 4]; Available from: https://pubchem.ncbi.nlm.nih.gov/compound/Redaporfin.
4. Information, N.C.f.B. PubChem Compound Summary for CID 5488036, Talaporfin sodium. [cited 2024 July 4]; Available from: https://pubchem.ncbi.nlm.nih.gov/compound/Talaporfin-sodium.
5. Information, N.C.f.B. PubChem Compound Summary for CID 32343, Rose Bengal. [cited 2024 July 4]; Available from: https://pubchem.ncbi.nlm.nih.gov/compound/Rose-Bengal-Sodium.
6. Information, N.C.f.B. PubChem Compound Summary for CID 6099, Methylene Blue. [cited 2024 July 4]; Available from: https://pubchem.ncbi.nlm.nih.gov/compound/Methylene-Blue.
7. Information, N.C.f.B. ubChem Compound Summary for CID 11967809, Cardio-Green. [cited 2024 July 4]; Available from: https://pubchem.ncbi.nlm.nih.gov/compound/11967809.
8. Inglut, C.T., et al., Predictors and Limitations of the Penetration Depth of Photodynamic Effects in the Rodent Brain. *Photochem Photobio*l, 2020. 96(2): p. 301-309.[PMID: 31441057 DOI: 10.1111/php.13155]
9. Rauf, M.A., et al., Solvatochromic behavior on the absorption and fluorescence spectra of Rose Bengal dye in various solvents. *Spectrochimica Acta Part A: Molecular and Biomolecular Spectroscopy*, 2009. 72(1): p. 133-137.[PMID: 18986828 DOI: 10.1016/j.saa.2008.08.018]
10. Baranovskii, S.F., P.A. Bolotin, and M.P. Evstigneev, Aggregation of 1,3,7-trimethylxanthine with methylene blue in aqueous solution. *Journal of Applied Spectroscopy,* 2006; 73(2): p. 171-177.[URL: https://citeseerx.ist.psu.edu/document?repid=rep1&type=pdf&doi=82e05cff9fe487542bdd3b1fea253bd38d4e88e4]
11. Yuan, B., N. Chen, and Q. Zhu, Emission and absorption properties of indocyanine green in Intralipid solution. *J Biomed Opt*, 2004; 9(3): p. 497-503.[PMID: 15189087 DOI: 10.1117/1.1695411]
